# Further Observations on “Trench Fever”

**Published:** 1918-01

**Authors:** 


***Further Observations on "Trench Fever".*** By G. H. Hunt and J. W. McNee. Abs. of the part of the article dealing with the louse problem. *Quarterly Journal of Medicine*, July, 1916, Vol. 9, No. 36.

The authors speak of an investigation to ascertain the cause of an outbreak of trench fever among the personnel of a clearing station. They paid special attention to the following points :

1. Had the men affected been nursing patients suffering from the disease, or had they been in contact with other men in the unit who developed it?

2. Had they been bitten by lice, or noticed them on their clothes?

3. Can a man have separate attacks of the disease?

All but 3 of the 13 men affected had been tending upon cases of trench fever. One of these three had slept with a man who had the disease, and he fell ill six days later than his bed companion. One of the other two was employed in handling the blankets of the hospital, and one was a stretcher-bearer.

Seven of the 13 men knew that they had been bitten by lice. The fact that the others had not noticed lice was not conclusive evidence that they had not been bitten.

The authors note 3 cases of late recurrence of the fever, 2 of which they think were certainly due to the virus of the original infection, but the other showed no such connection. Unless the late symptoms in this last case be considered a separate attack, it must be supposed that the virus remained completely dormant for 33 days.

The authors conclude that evidence has been obtained that the louse is the parasite transmitting the disease. This theory is further supported by the fact established by McNee and Renshaw, that the virus is contained in the corpuscular elements of the blood. They admit, however, that absolute proof is lacking, but they think that the indirect evidence obtained builds up a good case for the louse theory of transmission.

They argue for their conclusion as follows :

Personal contact alone seems insufficient for transmission, for three orderlies in a small ward of ten beds attending cases for several months were not infected. In cases where orderlies attending trench fever patients have contracted the disease they have worked in large wards with conditions making possible communication by lice.

When men who have not come personally in contact with patients have acquired the disease, they have been liable to lice infection. One was an attendant of the Thresh disinfector, one was the attendant of the incinerator, one was a stretcher-bearer, and one had charge of soiled blankets.

Seven of the 13 men who contracted the fever knew that they had had lice. Besides, the outbreak in the hospital force took place three weeks after the battle of Loos, when hundreds of sick and wounded were passed through rapidly with increased chances of louse infection.

Almost all the men coming to the hospital with the disease know that they have had lice. It is further noteworthy that officers and men are infected in almost equal proportions; this is also true of their infestation with lice.

The disease appeared first among men of only two classes, those who had been in the trench zone, and those in the Royal Army Medical Corps. Such men would naturally come first in contact with lice. The later spread of the disease away from the front may easily be explained by the increase of lice infestation elsewhere as the troops were transferred.

It does not appear that other insects were responsible. The disease persists unchecked throughout the winter making infection by flies or mosquitoes unlikely. Fleas are very rare among the troops.

Although this evidence is not complete or conclusive, it at least makes highly probable the transmission of trench fever by the louse.

